# The use of the nest for parental roosting and thermal consequences of the nest for nestlings and parents

**DOI:** 10.1007/s00265-017-2400-7

**Published:** 2017-11-07

**Authors:** Jan-Åke Nilsson, Andreas Nord

**Affiliations:** 1 Department of Biology, Section of Evolutionary Ecology, Lund University, Ecology Building, SE-223 62 Lund, Sweden; 2 Present Address: Department of Arctic and Marine Biology, Arctic Animal Physiology, Arctic Biology Building, University of Tromsø, NO-9037 Tromsø, Norway

**Keywords:** Body temperature, Heterothermy, Nest temperature, Reproductive cost, Roosting, Thermoregulation

## Abstract

**Abstract:**

At temperate latitudes, altricial birds and their nestlings need to handle night temperatures well below thermoneutrality during the breeding season. Thus, energy costs of thermoregulation might constrain nestling growth, and low nocturnal temperatures might require resources that parents could otherwise have invested into nestlings during the day. To manipulate parental work rate, we performed brood size manipulations in breeding marsh tits (*Poecile palustris*). Nest box temperatures were always well above ambient temperature and increased with increasing brood size. In line with predictions, a large majority of females (but no males) made use of this benign environment for roosting. Furthermore, females tending enlarged broods, thereby having to work harder during the day, reduced their body temperature at night. This might have reduced nocturnal energy expenditure. Our finding that a higher proportion of enlarged, as compared to control, females continued to use the nest box as roosting sites even after a simulated predation event despite increased vulnerability to predation, further highlighting the need for energy conservation in this group. High nest box attendance and reduced body temperature in brood-reduced females may indicate that these females prioritised self-maintenance by initiating other costly physiological adjustments, e.g. moult, when relieved from parental work. We suggest that the energy demand for defending homeothermy is an element of the general trade-off between current and future reproduction, i.e. between daytime investment in food provisioning and the potential short- and long-term costs of a reduction in body temperature and increased predation risk.

**Significance statement:**

Even during summer at temperate latitudes, breeding birds need to use energy to maintain stable body temperature. Parents, thus, need to enter the night with sufficient body reserves to cover energy requirements for thermoregulation. As these resources could be used for feeding nestling during the day, adaptations to reduce the cost of thermoregulation would be selected for. We performed brood size manipulations, thereby increasing the need for nestling provisioning in marsh tits (*Parus palustris*). We found that females typically spent the night in the thermally benign environment of the nest box together with their brood. Females working hard during the day continued to roost in the nest box during the night despite an increase in the perceived risk of nest predation. Furthermore, these females reduced their body temperature at night, thereby reducing the gradient between ambient and body temperature, further reducing the cost of thermoregulation.

## Introduction

In temperate areas, ambient night temperature is commonly below the thermoneutral zone (TNZ) of most birds throughout the year. Although strategies to handle such nocturnal thermal environments have been extensively studied during winter (Nilsson and Svensson 1996; McKechnie and Lovegrove 2002; Nord et al. 2009), almost no studies have investigated how birds handle the same problem during breeding. This is unfortunate, because nestling provisioning is among the most energetically costly activities of adult birds with altricial young (Drent and Daan 1980). This suggests that the added challenge of thermoregulation during cold nights may require resources that could otherwise have been invested in nestling provisioning during the day. Thus, to increase investment in parental effort, females might have to compromise self-feeding and the accumulation of fat for use during the night. To balance resource investment between somatic demands and nestling provisioning, females tending large broods (where energy expenditure during feeding is higher, Nilsson 2002) might have to reduce nocturnal body temperature, which is risky from a predation perspective (Carr and Lima 2013). Nestlings could also be affected by ambient temperatures below thermoneutrality, because they are energy-limited owing to their high growth rates (Ricklefs 1983). Thus, the extent to which adults and nestlings need to devote energy to thermoregulation should affect their breeding and fledging success, respectively.

Altricial birds lack thermoregulatory capacity in early life, and parents need to brood their young for a large part of the day shortly after hatching. The time invested in brooding decrease in line with the increased ability of the nestlings to thermoregulate (Alatalo et al. 1982). Nestlings of many species are functionally homeothermic from a relatively early age (e.g. Dunn 1975; Pereyra and Morton 2001; Węgrzyn 2013) owing to a combination of the development of endothermy and increasing thermal mass of the brood. Thus, nestlings may need to trade-off growth rate for thermoregulation to an extent which is dependent on the thermal environment of the nest (Andreasson et al. 2016). It is therefore not surprising that the thermal environment in the nest has proved important for fledging success (Ardia 2013; Dawson et al. 2005).

Parents feeding nestlings operate at a very tight energy budget (Drent and Daan 1980). If they also have to engage in thermogenesis during the night, they might need to trade off daily nestling feeding frequency against self-feeding to accumulate reserves to meet nocturnal demands for thermoregulation. Ambient temperatures below thermoneutrality during breeding could therefore be predicted to impair reproductive success by imposing energy stress on parents (cf. Tattersall et al. 2016). However, at least two options to mitigate the reduction in feeding frequency due to nocturnal thermoregulatory requirements are open for parents. They may (i) roost together with their young, thereby taking advantage of the warmer environment inside the nest (cf. Paquet et al. 2016), and/or (ii) reduce body temperature during the night. Both of these strategies result in a reduction of the thermal gradient between the body and the environment. The consequent reduction in metabolic heat loss and the lower metabolic demands of colder tissues then results in reduced energy requirements during the night (Cooper and Gessaman 2005). However, roosting inside the nest and/or reducing body temperature at night may potentially increase nocturnal predation risk, at least in cavity-nesting birds (Dunn 1977; Dhondt et al. 2010; Carr and Lima 2013; Nord et al. 2014; see also Grubb and Pravosudov 1994). Thus, the use of these adaptations can be predicted to increase with parental effort under the assumption that high parental effort restricts the time for self-feeding and reduces the possibility to store large energy reserves on the body. This could require hard-working females to trade-off predation risk (Dunn 1977) for roosting in a more thermally benign environment. In line with this, wintering blue tits (*Cyanistes caeruleus* L.) reduce resting body temperature to a larger degree when food is limited (Nord et al. 2009) and when they enter the roosting period with low fat reserves (Nord et al. 2011). Thus, when environmental conditions preclude the accumulation of sufficient reserves, birds may adopt physiological strategies to reduce nocturnal energy requirements, even when such strategies come at increased predation risk. Whether similar responses occur in small birds also during the breeding season is not known.

The aim of this study was to investigate trade-offs between thermoregulatory- and anti-predation behaviours in breeding marsh tits (*Poecile palustris* L.). We experimentally increased workload (by increasing feeding effort) during nestling provisioning by enlarging brood size. We predicted (i) that the nest environment during the night would be thermally more benign than outside the nest cavity, and that nest temperature would increase with brood size; (ii) that parents should make use of this environment for roosting; (iii) that the benefit of roosting in this thermally benign environment could be offset by increased perceived predation risk; (iv) that females with experimentally increased workloads would need to reduce nocturnal body temperature because they enter the night with smaller energy reserves due to restrictions in the time to self-feed during the day; (v) that this would further result in that these females being more dependent on a thermally benign roosting environment compared to females tending control or reduced broods and (vi) are therefore also more willing to accept higher predation risks for using such environments compared to females tending control or reduced broods.

## Methods

### General methods

The study was conducted during two breeding seasons, 2010 and 2011, in a nest box breeding population of marsh tits at Revingehed, 20 km east of Lund, southern Sweden (centred at 55° 42′ N, 13° 28′ E). The nest box area (64 km^2^) consists of small deciduous woods and groves surrounded by permanent pastures. Nest boxes were made of wood (thickness 2.2 cm), had an inner surface area of 7.8 × 9.5 cm, a height of 20 cm, an entrance hole with a diameter of 2.6 cm, and were erected about 1.5 m aboveground. In the beginning of the breeding seasons, the nest boxes were visited weekly to determine the day of the first egg (assuming that one egg is produced per day) and clutch size. Boxes were visited daily from the day before estimated hatching (incubation day 11; day of last egg = 1) to hatching to determine the exact day of hatching. Marsh tits are small (10–11 g), cavity nesting passerines, mainly found in deciduous woods. Females in the area produce a clutch of 5–11 eggs with yearly means usually between 7 and 9 eggs (Nilsson 1991). Females incubate alone and feed the nestlings together with the male for 19–21 days (Nilsson and Svensson 1993).

### Experiment

We experimentally altered brood size by adding or removing young from nests during both breeding seasons, thus creating enlarged and reduced broods with un-manipulated nests serving as controls. Manipulated broods were matched pair-wise with respect to hatching date and were chosen to minimise travel distance. Control broods were distributed evenly over the hatching date span of the manipulated broods. Six days after hatching, we moved three or four nestlings from the reduced to the enlarged broods, corresponding to a mean reduction of 44.3% (SD = 5.5) and a mean increase of 44.8% (SD = 7.4), respectively, in relation to the original brood size. This resulted in a significant (ANOVA *F*_2,92_ = 267; *P* < 0.001) difference between the sizes of experimental broods (*X* ± SD; reduced 4.28 ± 1.25, *N* = 39; control 7.84 ± 1.48, *N* = 46; enlarged 12.41 ± 1.24, *N* = 39). Nestlings in these broods, as well as those in control broods, were ringed and weighed on day 6. Part of the brood was first taken from the nest box to be ringed. These were then returned to the box and the second part was taken to be ringed and either returned to the same box (control) or to another box (enlarged). All ringing was performed out of sight from the nest box, and care was taken not to approach the box when parents were close to the box. This procedure ensured a low level of disturbance and ensured the same level of potential disturbance to all experimental categories.. Laying date and clutch size did not differ between the three categories of broods in any year of the study (ANOVA *P* > 0.15 in all cases). After manipulation, sample size was reduced due to predation of either the whole, or parts of, the brood, or of one of the parents. Furthermore, three control broods (with normal clutch sizes) that only hatched half the clutch due to unfertilized eggs were assigned to the reduced category and one enlarged brood that suffered from partial predation (thereby reducing the brood to its un-manipulated size) was assigned to the control category. Thus, the final sample size included 29 enlarged broods (2010 *N* = 13; 2011 *N* = 16), 37 control broods (2010: *N* = 19; 2011 *N* = 18) and 29 reduced broods (2010 *N* = 18; 2011 *N* = 11). It was not possible to record data blindly, because our study involved focal animals in the field.

At day 6 after hatching, we attached a small temperature data logger (iButton DS1922-L, Maxim integrated Products, Sunnyvale, CA; accuracy ± 0.5 °C) to the wall of the nest box, 1 cm above the nest rim (to ensure that the loggers were not within reach of nestlings or any roosting parent) to measure the thermal environment in a subset of the nest boxes. Sample size for nest box temperature was 38 in 2010 and 41 in 2011 (both years combined: enlarged *N* = 27; control *N* = 27; reduced *N* = 25). The data logger recorded nest box temperature with a sampling interval of 4 min and a resolution of 0.0625 °C. For the analyses, we retrieved temperature data as the mean for the period 02.00–04.30 during the night after day 9 or 10, because these nights coincided with our night-time measurements of female body temperature (see below). Ambient temperature was measured with the same sampling interval and resolution in the shade at a sheltered place (1.5 m above ground) in the middle of the study area.

We searched nest boxes for roosting parents at night when nestlings were 9 or 10 days old, at the time when daytime feeding rates are at their peak (J-ÅN and AN, unpublished) coinciding with maximum growth rates of the nestlings (Perrins 1979). During this period, we found all but two females, both in 2011, in their nest box. To measure body temperature, we used a Testo 925 digital thermometer (Testo AG, Lenzkirch, Germany) equipped with a type K (chromel-alumel) thermocouple (Ø = 0.9 mm; ELFA AB, Järfälla, Sweden) calibrated by an accredited calibration laboratory (Nordtec Instrument AB, Göteborg, Sweden). The thermocouple was inserted 12 mm into the cloaca (further insertion did not alter the temperature reading). When we opened the nest box roof, females were still sleeping and the time between capturing the female and inserting the thermocouple was less than 5 s. We then obtained three body temperature readings with the thermocouple in place within the next 5 s [inter-sample repeatability; 2010 *r* = 0.99; *N* = 50 females; *P* < 0.001; 2011 *r* = 0.99; *N* = 43 females; *P* < 0.001 (Lessells and Boag 1987); average used in analyses]. All temperature measurements were conducted between 23:10 and 01:50 (i.e. 2 to 4.5 h after sunset). After the temperature measurement, we aged the females according to Svensson (1992), or if already ringed (63%) according to our previous age record of the female. Previously unknown females that were aged as older than in their second calendar year (*N* = 36) were assigned the age of 3 years. We then returned the female to the box. All females settled in the box immediately upon return, and none of them left the box during the night.

As the first nightly visit to the nest box would probably be perceived as a predation attempt (cf. Nord et al. 2014), a subset of the nest boxes (enlarged broods *N* = 19; control broods *N* = 15; reduced broods *N* = 11) were visited a second night, one or two nights after the initial measurement, to assess if females changed their roosting behaviour in response to a perceived increased predation risk. Those females found roosting in the nest box during the second visit (enlarged broods *N* = 17; control broods *N* = 8; reduced broods *N* = 9) were sampled for body temperature a second time (only the first measure used in analyses of the experimental effect; see below). To determine how the experiment affected adult condition, we recaptured females during nestling feeding on day 14 to measure their body mass (± 0.1 g) and tarsus length (± 0.1 mm). We measured nestling body mass at the same occasion and for the same reason.

### Statistical procedures

Statistical analyses were performed using SAS Enterprise Guide 4.3 (SAS Institute Inc., Cary, NC, USA). When investigating factors determining the thermal environment in the nest box during the night, we included ambient night temperature and brood size as covariates, experimental category (enlarged, control or reduced brood), year, and night of measurement (nestling day 9 or 10) as fixed factors in the initial model. To test which variables could explain a significant part of the variation in female night-time body temperature, we used a mixed effects model with female identity as the random variable because 13 females were included in both years. In the initial model, we included nest box night temperature, female age (2 to 7 years), and brood size as covariates, and experimental category (enlarged, control or reduced brood), year, and night of measurement (nestling day 9 or 10) as fixed factors. The model also included the interactions between experimental category and year, brood size, and nest box night temperature, respectively, and between box night temperature and brood size. To explain variation in female mass at nestling day 14, we included experimental category and year as fixed factors, and female tarsus length as a covariate, in a mixed model with female identity as a random factor. We also tested predictors of mean nestling mass at day 14 by including experimental category and year as fixed factors, and female condition (i.e. the residual mass from the relationship between mass and tarsus length of the females) as a covariate, in a mixed model with female identity as a random factor. To explain variation in the frequency of nest box roosting the night after a disturbance, we used a binomial mixed model with roosting (yes/no) as the dependent variable and included year and experimental category as fixed factors, nest box temperature as a covariate, and female id as a random factor. All mixed models were fitted using the residual maximum likelihood method (REML), and initial models were reduced by backward elimination of non-significant variables until only significant terms (*P* < 0.05) remained. Due to collinearity between brood size and experimental category, in final models containing one of these terms, we exchanged the selected one with the other and re-ran the model. We then evaluated these two models using the Akaike Information Criterion (AIC) and selected the model with the lowest AIC value as the final model. To be able to compare alternative final models, these were fitted using maximum likelihood (ML) instead of REML (Littell et al. 2006). Denominator degrees of freedom were calculated using the Satterthwaite approximation. All significance estimates are from two-tailed tests.

## Results

The brood size manipulation did not affect female mass at nestling day 14 (ANOVA *F*_2,73.6_ = 0.80; *P* = 0.45), but females with longer tarsi were heavier (*F*_1,79.5_ = 7.81; *P* = 0.0065). However, mean nestling mass at day 14 was affected by the experiment (*F*_2,83_ = 6.86; *P* = 0.0017). Specifically, nestlings from enlarged broods (*X* ± SE = 11.3 ± 0.15 g) were significantly lighter than nestlings from control (11.8 ± 0.13 g) and reduced (12.0 ± 0.16 g) broods (Tukey’s post-hoc test: *P* = 0.042 and *P* = 0.0013, respectively). Nestling mass did not differ between control and reduced broods (*P* > 0.3). Nestling mass was also positively affected by female condition (*F*_1,83_ = 5.30; *P* = 0.024), and nestlings were heavier in 2011 (*X* ± SE = 12.0 ± 0.12 g) than in 2010 (11.4 ± 0.12 g) (*F*_1,83_ = 13.8; *P* = 0.0004). The difference between years was further emphasised by nestling mortality, which was higher in 2010 (total 7.2%; enlarged 10.5%; control 6.7%; reduced 0%) than in 2011 (total 0.5%; enlarged 0%; control 1.3%; reduced 0%).

**Fig. 1:**
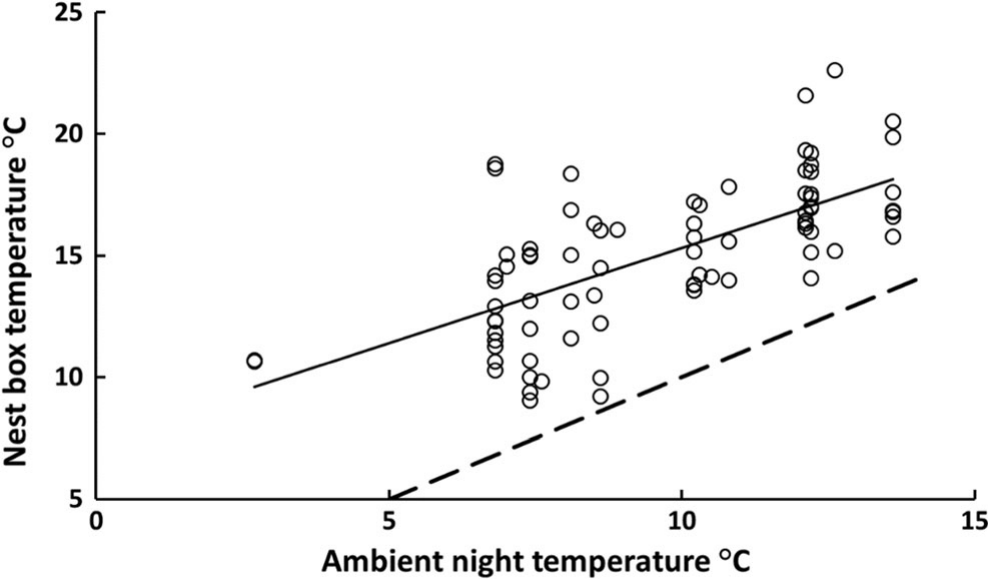
The relationship between ambient night temperature and temperature within the nest box containing 9–10 days old marsh tit nestlings (*N* = 79 independent broods). Equation of the line: *Y* = 0.79*x* + 7.5; *P* < 0.001; *R* ^2^ = 0.45. Broken line denotes conditions when ambient night temperature and temperature within the nest box is the same

**Fig. 2:**
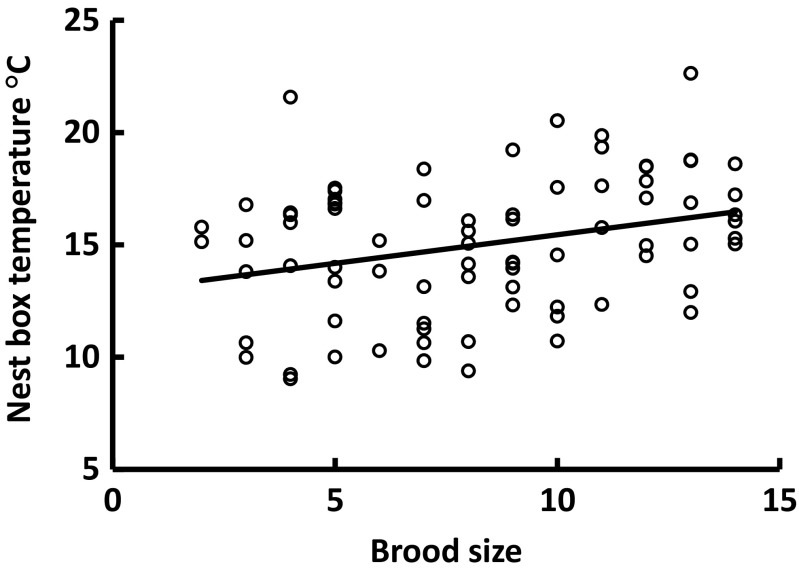
The relationship between brood size and temperature within the nest box containing 9–10 days old marsh tit nestlings (*N* = 79 independent broods). Equation of the line: *Y* = 0.25*x* + 12.9; *P* = 0.008; *R* ^2^ = 0.09

The insulating properties of the nest and nest box, and the presence of nestlings, resulted in the nest box environment being 4–7 °C warmer than ambient night temperature (mean difference ± SE: 5.41 ± 0.26 °C; paired-sample *t* test: *t*_78_ = 20.5; *P* < 0.001). Nest box temperature was positively affected by both the ambient temperatures (*F*_1,73.5_ = 137.4; *P* < 0.0001; Fig. 1) and the number of nestlings, with each additional nestling resulting in an increased nest box temperature of 0.25 °C (*F*_1,75.7_ = 51.0; *P* < 0.0001; Fig. 2). To test if experimental category would be as good a predictor as brood size for night-time nest temperature, we exchanged these two variables and re-ran the model (with ML parameter estimation) and used AIC to select the best fitting model. Although experimental category could also explain a significant part of the variation in nocturnal nest box temperature (*F*_2,69.1_ = 23.8; *P* < 0.0001), our initial model containing brood size provided a significantly better fit to data (ΔAIC = 7.0). Thus, it was the number of nestlings per se and not any consequences of the manipulation (e.g. increased variation in nestling growth rate or parental work rate) that best determined the thermal environment within the box. In summary, the thermal environment for the nestlings depends on ambient night temperature and the number of brood mates.

**Fig. 3:**
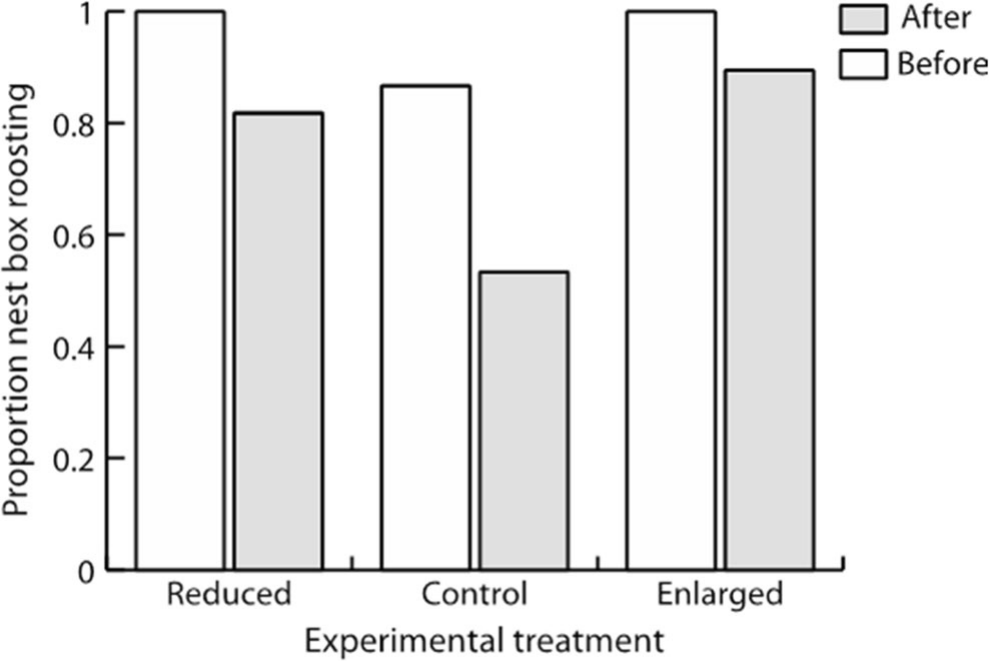
Proportion of female marsh tits in the experimental treatments, roosting in their breeding nest box before (enlarged *N* = 29, control *N* = 37, reduced *N* = 29) and after (enlarged *N* = 19, control *N* = 15, reduced *N* = 11) a nocturnal disturbance. The disturbance was a visit to measure the body temperature of the female. When this was done for the first time, it is denoted “before”, and when it was done a second time, 1 or 2 days later, it was denoted “after”. Brood sizes were manipulated to be either enlarged or reduced, with un-manipulated broods serving as controls

During the first visit to a nest box, most females (98%) roosted together with their brood. Only two females, both of which reared control broods, roosted elsewhere. However, we did not find a single male roosting in the nest boxes. Moreover, females that still found roosting in the nest boxes during the second visit were not evenly distributed among the experimental categories (*F*_2,32.9_ = 3.48; *P* = 0.043). Only 8 out of 15 (53%) control females continued to roost in the nest after being disturbed on a previous night, whereas 17 of 19 (90%) and 9 of 11 (82%) females with enlarged and reduced broods, respectively, did so (Fig. 3).

**Fig. 4:**
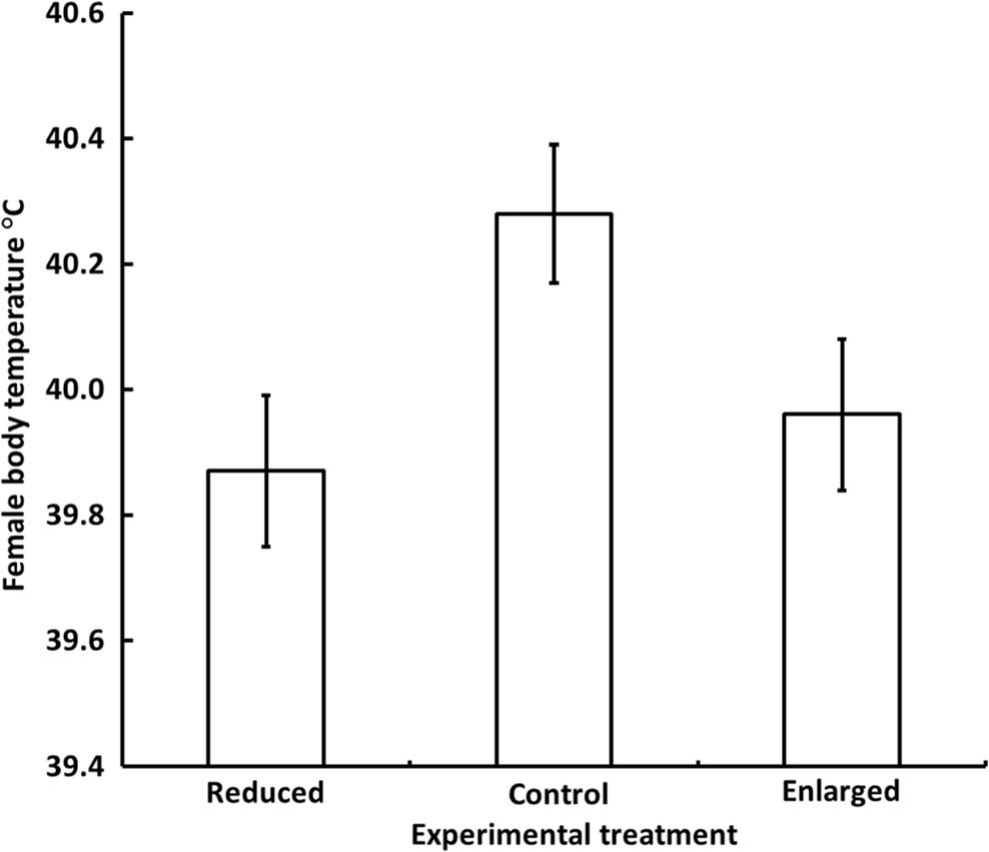
Estimated mean (± SE) female marsh tit body temperature during the night when roosting among her young (aged 9–10 days) in a nest box (enlarged *N* = 29, control *N* = 37, reduced *N* = 29). Brood sizes were manipulated to be either enlarged or reduced, with un-manipulated broods serving as controls. Tukey’s HSD tests: enlarged vs. control *P* = 0.036; reduced vs. control *P* = 0.008; enlarged vs. reduced *P* = 0.6

Body temperature of females measured during two different nights proved to be significantly repeatable (between-night repeatability; *r* = 0.35; *N* = 34; *P* = 0.019). Only one of the explanatory variables could explain any of the variation in female body temperature, viz. experimental category (*F*_2,44_ = 4.55; *P* = 0.016). Specifically, females roosting together with control broods had significantly higher body temperatures (*X* ± SE = 40.28 ± 0.11 °C) than females roosting together with both reduced (39.87 ± 0.12 °C) and enlarged broods (39.96 ± 0.12 °C; Fig. 4). Also here, we tested the interchangeability of experimental category and brood size by re-running the model (ML estimation) followed by AIC comparison. In this case, the experimental category model was a better fit to data than the brood size model (ΔAIC = 6.2). Thus, the body temperature of roosting females during the night is not primarily determined by the number of nestlings, but more by the additional factors connected to a manipulated brood size, e.g. increased variation in parental work load during daytime feeding.

## Discussion

Our brood size manipulation did not affect female body mass, at least not during the day, but resulted in a lower mean nestling body mass in enlarged broods. Thus, parents tending enlarged broods were not able to increase feeding rate sufficiently to compensate for increased sibling competition. Furthermore, parents tending reduced broods did not seem to increase investment into feeding effort as nestling body mass did not differ between control and reduced broods. Although feeding frequency was not measured in the present study, previous brood size manipulations in the same species and study population have resulted in significantly increased and reduced feeding frequencies in enlarged and reduced broods, respectively (Nilsson 2002).

Ambient night temperatures at our field site varied between 2.7 and 13.6 °C, thus always being below TNZ of the marsh tit (around 22 °C among similar sized tits in summer; Gavrilov and Dolnik 1985; Gavrilov 1999). The nest box, with its nest and brood, offered a much more benign nocturnal thermal environment, with a temperature that was on average 5.41 °C above ambient. Still, the temperature in the nest box was generally in a temperature range where thermogenesis was needed to defend a constant body temperature. Larger broods improved the thermal environment experienced by roosting females (Fig. 2), as each additional nestling increased nest box temperatures by 0.25 °C. Accordingly, nestlings in small broods must allocate more energy to thermoregulation than those in large broods or, alternatively, might require more brooding by the female; an effect that is further exacerbated by the larger surface area to volume ratio and reduced thermal mass in small broods (Mertens 1969). It is intriguing to speculate that the increase in clutch size with latitude, traditionally explained by a more pronounced seasonality in food resources (Ashmole 1963), reduced nest predation rate (Skutch 1949), or longer days during breeding (Lack 1947; Rose and Lyon 2013) in the north, could also be due to thermal characteristics in the nest (cf. discussion in Nord and Nilsson 2012). In cold northern environments, a large brood would decrease the thermoregulatory costs of nestlings (Andreasson et al. 2016), and on warm southern latitudes, a small brood would reduce the risk of overheating (cf. Ardia 2013).

At the time of our temperature measurements, individual nestlings were likely able to thermoregulate to some extent (Węgrzyn 2013; Andreasson et al. 2016), and the brood as a unit should have been functionally homeothermic since a few days (Pereyra and Morton 2001), even in experimentally reduced broods (Andreasson et al. 2016). In a study of the development of homeothermy, individual blue tit nestlings were found to reach a homeothermic index of 0.8 (considered to be the threshold for whether a nestling is homeothermic or not; Visser 1998) at a nestling age of 8 days (Andreasson et al. 2016). In line with this, daytime brooding decreased sharply when great tit (*Parus major* L.) nestlings were 6 days old (Sanz and Tinbergen 1999). Thus, we believe that the presence of the females in the nest boxes was not primarily needed for promoting nestling thermal balance. This was further emphasised by the fact that a majority (24 out of 43 occasions were the female position in the nest was recorded) of the females were roosting beside the brood in minimal contact with the nestlings. Furthermore, none of the females adopted a brooding-like behaviour, i.e. trying to cover the nestlings, but were merely sitting on top of the nestlings (own observations). Instead, we suggest that females choose to roost with their brood mainly to exploit thermal benefits of the nest environment, which could allow for a reduction of their own nocturnal energy expenditure. In addition, their presence in the nest box will further improve the thermal environment by at least as much as one additional nestling, i.e. by 0.25 °C. Assuming a lower critical temperature (LCT) of 22 °C (Gavrilov and Dolnik 1985; Gavrilov 1999) and a linear increase in energy consumption with decreasing ambient temperatures below LCT (Gavrilov 1999), calculations from previous studies of great and blue tits in the same study area (Nilsson and Svensson 1996; Broggi et al. 2004), roosting in the 5 °C warmer environment of the nest box should have reduced female metabolic rate by about 10%. This energetic benefit might come at a cost of increased predation risk from nocturnal, mammalian predators (Dunn 1977). However, potential predation risk seems to be outweighed by the advantages of reducing nocturnal energy expenditure in our population (cf. Nord et al. 2014), because 98% of the females spent the night together with their broods. Interestingly, this option does not seem to be open for males. Accordingly, we would predict that males must self-feed to a larger extent than females to build up larger reserves for the night, with reduced nestling feeding rates as a potential consequence. In line with this, previous studies at the same study site report that female marsh and blue tits have a slightly higher (although not significantly so) daily feeding frequency than males (i.e. 54% of all feeding visits were made by the female; Nilsson 2003; Råberg et al. 2000). However, to minimise energetic flight costs, the reserves for the night should be accumulated as late in the day as possible (Norberg 1981). Thus, we predict that sex differences in feeding frequency should be most pronounced during late afternoon.

Significant energy savings can be achieved by not defending homeothermy during the night, especially at temperatures close to LCT (Cooper and Gessaman 2005; Brodin et al. 2017). This should be most important for hard-working individuals that have fewer reserves available to cover the energy cost of thermoregulation but still may have to sustain an upregulated BMR (Nilsson 2002; Tieleman et al. 2008; Careau et al. 2013). In line with this, females caring for enlarged broods reduced their body temperature more than females tending control broods (Fig. 4), in spite of a possible increased heat production due to a higher BMR. The estimated mean nocturnal body temperature of brood-enlarged females was 39.96 °C, which is considerably lower than in same-sized blue tit females (41.9 °C) during winter nights when food is available ad libitum or in blue tits during the day (42.6 °C; Nord et al. 2009). Thus, it seems as if females tending enlarged broods do not defend a normothermic nocturnal body temperature during the breeding season. The fact that experimental brood size categories explained variation in data better than the number of nestlings indicated that the degree to which females of enlarged broods reduced body temperature was determined by the manipulated increase in work rate per se. In line with this, birds experimentally made to work harder have been found to reduce night-time resting metabolic rate (Wiersma and Tinbergen 2003), which could be achieved by regulating nocturnal body temperature to a lower set point. Based on the relationship between metabolic rate and reductions in nocturnal body temperature in other studies (McKechnie and Lovegrove 2003; Tattersall et al. 2016), the lower body temperature in brood-enlarged females in our study may have ameliorated nocturnal metabolic rate by 3.6–8.6%. The ecological and physiological significance of this reduction should be addressed in subsequent studies.

The general importance of energy conservation at night following hard daytime work is illustrated by the observation that females feeding enlarged broods were largely unaffected by a previous simulated predator attack (i.e. our first visit to the nest box). In contrast, nearly half of the control females decided to roost elsewhere on the night after disturbance. It should be noted that our study area consists of managed forest patches with young trees, resulting in very few natural cavities. Therefore, the vast majority of the marsh tits breed, and roost, in nest boxes. Thus, the need to save energy during nights may be decisive for the outcome of the trade-off between predation risk and need for thermoregulation (cf. Nord et al. 2014; Todd et al. 2016) in females tending enlarged broods.

Females tending reduced broods also maintained a lower nocturnal body temperature than did control females. Moreover, these females (like those caring for enlarged broods) were more prone to return to the nest box on subsequent nights despite a previous simulated predator attack. These observations point to increased need, or use, of behavioural strategies for energy conservation. However, they do not explain *why* brood-reduced females adopted such strategies to a larger degree than control females. It seems unlikely that this was a result of compensation for increased parental effort, because these females did not produce significantly heavier nestlings than control females and parental feeding frequency has been shown to be substantially reduced in females tending reduced broods in comparison to control females in the same species (Nilsson 2002). Furthermore, it can be argued that the brood size manipulation per se could have affected female body temperature. However, since parents of all three categories were disturbed to the same extent, we do not know of any experimental design-related mechanism that could differentially affect females of enlarged and reduced broods as compared to those of control broods. Instead, we propose that brood-reduced females might have behaviourally (and physiologically) tighter nightly energy budgets due to engaging in other energetically costly activities. (1) It is possible that increased metabolic rate in these females was a result of an earlier start of the physiological process of moulting, because parents working less may advance their start of moulting into the nestling feeding period (Morales et al. 2007). This may relieve time constraints on moulting into a high-quality plumage (cf. Nilsson and Svensson 1996; Broggi et al. 2011). Because moult is energetically costly (Lindström et al. 1993), females tending reduced broods in our study might have reduced body temperature and prioritised risky nest box roosting to reduce thermoregulatory costs at night when the cost of moult is most pronounced (Cyr et al. 2008). (2) Related to this explanation would be that males might reduce their feeding effort in small broods, resulting in a need for female compensation. In analogy, males often start to moult before their mates and moulting males have been found to reduce their feeding frequency (Svensson and Nilsson 1997). (3) Another mechanism with the potential to affect physiological processes is stress. Brood-reduced females might have perceived the reduction in brood size as an act of nest predation, which in turn could have increased levels of stress hormones, e.g. corticosterone (Clinchy et al. 2004). In line with this, birds that were implanted with corticosterone implants were found to be less readily disturbed and had lower metabolic rate during night than sham-implanted ones (Buttemer et al. 1991). Both the reduced metabolic rate and the nocturnal decrease in responsiveness as a result of higher stress hormone levels are compatible with a reduced body temperature. However, further studies are needed to elucidate the energy allocation strategies employed by parents suddenly relieved of some of their parental effort.

In conclusion, our study has shown that the, largely ignored, demand on energy allocation to thermoregulation during the breeding season at temperate latitudes might affect the energy balance of both parents and nestlings. This results in a trade-off between energy expenditure for thermoregulation and predation risk, as indicated by females accepting an increased perceived risk of predation to reduce the energetic cost of thermoregulation. Furthermore, females feeding large broods might have less time available for putting on reserves for the night, which might come at the cost of reduced nocturnal body temperature. Also, females feeding reduced broods reduced body temperature during night. This might be explained either by the manipulation procedure per se or by the reduced brood size initiating other energy demanding processes in these females. The fact that year did not explain any of the variation in female behaviour in spite of large variation in nestling mortality and nestling mass suggests that our results seem to be representative for a broad range of circumstances. A better understanding of these relationships will add important insights into the general trade-off between current and future reproduction.
